# Metabolomics as a powerful tool for diagnostic, pronostic and drug intervention analysis in COVID-19

**DOI:** 10.3389/fmolb.2023.1111482

**Published:** 2023-02-15

**Authors:** Chiara Bruzzone, Ricardo Conde, Nieves Embade, José M. Mato, Oscar Millet

**Affiliations:** ^1^ Precision Medicine and Metabolism Laboratory, CIC bioGUNE, Basque Research and Technology Alliance (BRTA), Bilbao, Bizkaia, Spain; ^2^ CIBERehd, Instituto de Salud Carlos III, Madrid, Spain

**Keywords:** COVID-19, SARS-CoV-2 infection, metabolomics, lipidomics, NMR, LC/GC-MS, phenoreversion, long covid

## Abstract

COVID-19 currently represents one of the major health challenges worldwide. Albeit its infectious character, with onset affectation mainly at the respiratory track, it is clear that the pathophysiology of COVID-19 has a systemic character, ultimately affecting many organs. This feature enables the possibility of investigating SARS-CoV-2 infection using multi-omic techniques, including metabolomic studies by chromatography coupled to mass spectrometry or by nuclear magnetic resonance (NMR) spectroscopy. Here we review the extensive literature on metabolomics in COVID-19, that unraveled many aspects of the disease including: a characteristic metabotipic signature associated to COVID-19, discrimination of patients according to severity, effect of drugs and vaccination treatments and the characterization of the natural history of the metabolic evolution associated to the disease, from the infection onset to full recovery or long-term and long sequelae of COVID.

## Introduction

Undoubtedly, COVID-19 outbreak emerged as one of the biggest medical challenges worldwide. The virus responsible for COVID-19 is the Severe Acute Respiratory Syndrome Coronavirus 2 (SARS-CoV-2), named after SARS-CoV for its genetic similarity (Velavan and Meyer, 2020). Shortly after its first detection in Wuhan (China) in December 2019 the virus quickly spread out, and the World Health Organization (WHO) declared a state of pandemic in March 2020 (Cucinotta and Vanelli, 2020). Now a days the virus has caused more than 624 million infections and 6.5 million deaths^1^. SARS-CoV-2 is very easily transmitted from person to person through droplets inhalation together with aerosol emission and consequent contact of the virus with nose, mouth or eyes mucous (Ciotti et al., 2020; Esakandari et al., 2020), providing an explanation for the high virulence associated with this pathogen.

At first, COVID-19 was characterized primarily as a lung disease since the main symptoms targeted the respiratory track. Indeed, infection of SARS-CoV-2 starts by binding the virus’ spike protein to the angiotensin-converting-enzyme 2 (ACE2) receptor in the host cells surface (Li et al., 2003). ACE2 is principally expressed into the nasal epithelium cells and in the bronchial epithelia (Sungnak et al., 2020). Yet, ACE2 receptor is also present in other organs, providing a mechanistic explanation for the onset of secondary issues in other organs like renal insufficiency, heart or nervous system (Esakandari et al., 2020; Ashraf et al., 2021).

As any infectious disease, COVID-19 immediately triggers the immunological response, which is highly variable among patients and it depends on personal characteristics and different involved risk factors (Jackson et al., 2022). For a subset of patients, an excessive immune response (*cytokine storm*) is associated with a severe disease phenotype (Coperchini et al., 2020; Rothan and Byrareddy, 2020). Associated to it, the related acute respiratory distress syndrome (ARDS) consists in an increased release of immune system cells that has been associated with organ failure. Therefore, the combination of the cytokine storm and ARDS are considered the main causes of death among patients (Montazersaheb et al., 2022). Any case, the interplay between SARS-CoV-2 infection and the host immunological system largely regulates the disease progression and is ultimately responsible for the severity of the disease (Gallo Marin et al., 2021).

Altogether, considering the highly variable phenotypic response and the systemic character of the disease, it becomes clear the need of investigating COVID-19 using alternative techniques such as the ones used in precision medicine. The aim of precision medicine is to provide an individualised solution to health problems that a subject can present (Bissonnette and Bergeron, 2012; König et al., 2017). To account for it, the “omics” technologies can help building a suitable description of the individual’s specific characteristics to find personalized treatment for the cure of specific diseases (Zhang, 2015). Genomics, transcriptomic, proteomics and metabolomics investigated on non-invasive fluid samples (i.e., serum, plasma or urine) can provide useful information to understand the disparate response to a given disorder from different subjects (Ward et al., 2021). In this regard, metabolomics is specially suitable because it is more sensitive to any phenotypic alteration (Nicholson, 2021). Thus, metabolomics has been extensively applied to COVID-19 to provide mechanistic information of the disease, to find robust diagnostic and prognostic biomarkers and to investigate the natural history course of the infection. The present review aims to discuss all the advances in the knowledge of COVID-19 disease as provided by the metabolomic analysis of patient samples.

## An overview of metabolomic studies in COVID-19

As expected, COVID-19 has been extensively investigated using metabolomics approaches. We scrutinized the databases (Web of Science, Scopus and Pubmed, 2020–2022), using general keywords for a maximum coverage (Table 1). The outcome shows a very large number of contributions, in proportion with the emergency produced by the pandemic. After manually curating the results (Table 1), we found 90 search items that correspond to original contributions where metabolomics has been applied to COVID-19 patient cohorts. Only studies involving humans have been considered. All these studies involved either mass spectrometry coupled to gas chromatography (GC-MS) or liquid chromatography (LC-MS) (70%) and/or nuclear magnetic resonance (NMR) experiments, except for one (Robertson et al., 2022) that used Raman spectroscopy to investigate urine samples. LC-MS and GC-MS show an exquisite sensitivity that enables monitoring the entire metabolism with little signal overlapping but at the cost of limited reproducibility (Pan and Raftery, 2007). Additionally, they may require sample derivatization of the results and the quantification requires the use of standards (Bingol, 2018). In turn, NMR spectroscopy is highly complementary since it is fully quantitative, requires no derivatization and it is very reproducible, but with low sensitivity, which largely limits the accessible metabolome that can be investigated by this technique (Bruzzone et al., 2021). Remarkably, some contributions correspond to the development of new methodology for the application of these techniques to COVID-19 (Holmes et al., 2021b; Lodge et al., 2021c; Nitschke et al., 2022a; 2022b) or ring studies between laboratories (Masuda et al., 2021).

**TABLE 1:** Summary of the database searches as consulted in 17 November 2022.

	Web of science				PubMed				Scopus			
Search	All	2020	2021	2022	All	2020	2021	2022	All	2020	2021	2022
COVID and metabolom^*^	403	59	167	177	336	36	140	160	421	45	186	190
COVID and lipidom^*^	106	13	56	37	104	16	56	36	112	11	58	43
Curated	90 original contributions (not reviews nor clinical trials)											

^*^ means that has to search for any term that initiates by that word.

In terms of the nature of the specimen, serum and plasma followed by urine (Kurano et al., 2022b; Kida et al., 2022) are the most studied biofluids, while other samples also scrutinized include saliva (Spick et al., 2022b; Frampas et al., 2022; Saheb Sharif-Askari et al., 2022), faeces (He et al., 2021; Lv et al., 2021; Ren et al., 2021), platelets (Schuurman et al., 2022), exhaled breath (Barberis et al., 2021a; Grassin-Delyle et al., 2021; Bennet et al., 2022; Remy et al., 2022), sebum from skin (Spick et al., 2021) and breastmilk (Zhao et al., 2020). Due to the high volume of information (Table 1), we here complement other recent reviews on COVID-19 metabolomics (Hasan et al., 2021; Lin et al., 2021; Costanzo et al., 2022) by selecting a set of contributions to be discussed in the present review, based on our evaluation of novelty, impact and originality.

## Metabolic signature of the acute phase of SARS-CoV-2 infection

In addition to the consubstantial symptomatology of COVID-19, very early studies corroborated the fact that SARS-CoV-2 infection results in a much-altered metabolism, as determined from serum samples of hospitalized patients (Shen et al., 2020; Thomas et al., 2020). This metabotype is characteristic and differs from the one observed in flu-induced ARDS patients (Lorente et al., 2021). Significant alterations in the kynerurate/tryptophan pathway and abnormal glucose levels were amongst the first proposed metabolic markers associated to COVID-19 (Shen et al., 2020). These observations were further confirmed by other studies (Blasco et al., 2020; Bruzzone et al., 2020; Kimhofer et al., 2020; Song et al., 2020) that also overcame the technical limitations from the early cohorts (i.e., reduced size of the cohorts, sample inactivation and non-fasting conditions). Elevated kynurenic acid is gender-specific (Cai et al., 2021) and, in conjunction with other gender sensitive metabolites (Escarcega et al., 2022), it provides a rationale for the poorer clinical outcome in males than in females. Tryptophan is an essential amino acid and a neurotransmitter, and with phenylalanine also involved in the modulation of the immune response (Fernstrom and Wurtman, 1971) and inflammatory processes in lung and kidney diseases, or other infections like HIV or sepsis (Darcy et al., 2011; Kimhofer et al., 2020). SARS-CoV-2 infection also alters many other metabolic pathways (Albóniga et al., 2022), with changes in the serum amino acid signature (for instance, elevated glutamine/glutamate and Fischer’s ratios) (Doğan et al., 2021; Páez-Franco et al., 2022). Glutamate alteration in COVID-19 is in part mediated by the α-glutathione S-transferase, associated with certain processes such as liver failure, skeletal muscle metabolism, cancer or immunodeficiency (Kinscherf et al., 1996; Cruzat et al., 2018). Other found alterations include the circulating exosome (Alzahrani et al., 2021; Lam et al., 2021), the serum fatty acids (Chen et al., 2022) and other serum lipids such as carnitines (Castañé et al., 2022), ceramides (Dei Cas et al., 2021; Khodadoust, 2021) and phospholipids (Barberis et al., 2020; Shen et al., 2020; Janneh et al., 2021; Masoodi et al., 2022). The serum lipoprotein composition is also largely dysregulated (Bruzzone et al., 2020; Kimhofer et al., 2020; Lodge et al., 2021c; 2021a; Gray et al., 2021; Masuda et al., 2021), showing a pathogenic redistribution of the lipoprotein particle size and composition to increase the atherosclerotic risk.

**FIGURE 1:**
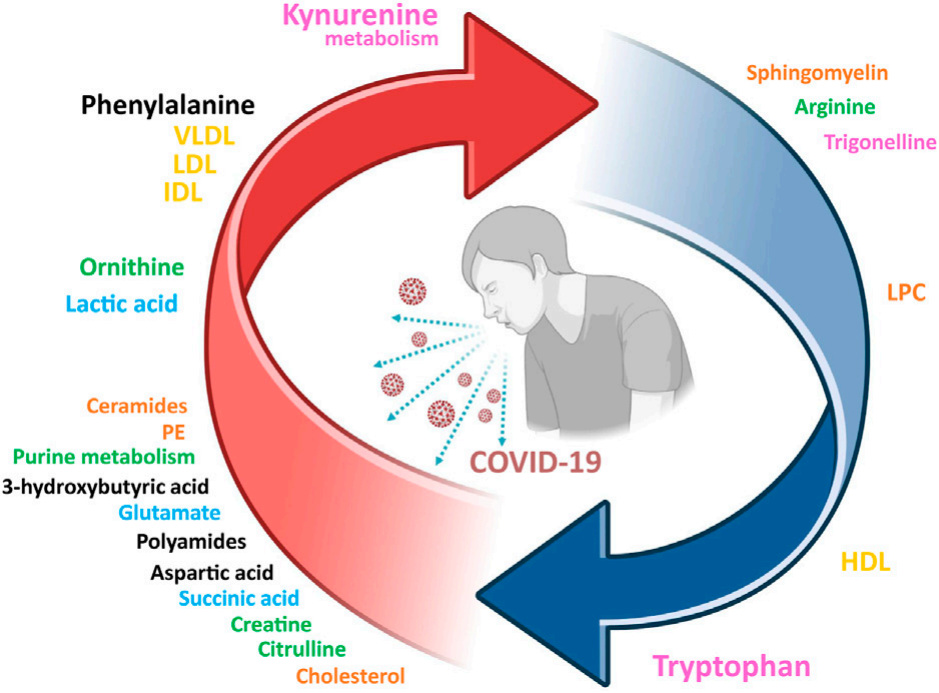
A consensus view of the metabolic signature of COVID-19 patients. Arrow colors indicate if metabolites are upregulated (red) or downregulated (blue). The letter size is proportional to the supporting experimental evidence. Color legend for the metabolic pathways: tryptophan metabolism (pink), central metabolism (blue), purine metabolism and urea cycle (green), lipids (orange), lipoproteins (yellow), other (black). VLDL, very low-density lipoproteins; LDL, low density lipoproteins; HDL, high density lipoproteins; IDL, intermediate density lipoproteins; PE, phosphatidylethanolamine.

Several studies used metabolic models to discriminate with great success (AUROC >0.95) between COVID-19 patients and healthy individuals (Bruzzone et al., 2020; Ruffieux et al., 2022). A simplified COVID-19 metabolic signature of the serum/plasma metabolomic dysregulation in acute patients can be obtained from the integrative analysis of the abovementioned studies, and it is shown in Figure 1. Undoubtedly, the strongest metabolic signal associated to SARS-CoV-2 is tryptophan metabolism (pink in Figure 1), whose catabolysis is upregulated with accumulation of kynurenine and other intermediates and depletion of trigonelline in COVID-19 patients. Related to this, upregulation of purine metabolites and some components of the urea cycle (blue in Figure 1) are also consubstantial to COVID-19. The increase in lactate and the dysregulation of essential metabolites in the central metabolism suggests a mitochondrial impairment. Finally, dyslipidemia is observed at the lipid level, with some lipids consistently showing upregulation (ceramides, PE, cholesterol) or downregulation (sphingomyelin and LPC), but also in the serum lipoproteins composition (yellow in Figure 1). All these studies are consistent with a model in which SARS-CoV-2 infection induces damage in the liver, the kidney, and other organs during the acute phase, also associated with dyslipidemia and oxidative stress.

This metabolic signature seems to be rather homogeneous worldwide, with equivalent results obtained when considering very distant geographical and cultural regions such as Mexico (López-Hernández et al., 2021), Italy (Saccon et al., 2021; Ghini et al., 2022b; Ciccarelli et al., 2022), and Africa (Li X. et al., 2022). Instead, characteristic but distinct metabotypes can be associated to pregnant women (Id et al., 2022), newborn (Kontou et al., 2021) and infantile (Wang et al., 2021) COVID-19 population. Finally, the COVID-19 metabotype changes concertedly with other subcellular elements (i.e., proteins, gene expression), that can be integrated to obtain the trans-omic landscape of COVID-19 (Wu et al., 2021).

Finally, COVID-19 is associated with an exacerbated inflammatory response and many studies have investigated the associated metabolic changes in conjunction with inflammatory markers (Lodge et al., 2021a; Yang et al., 2022). Specifically, IL-18, IL-6, IFN-γ, IP-10 and RANTES exhibited strong positive correlations with the pro-atherogenic LDL sub-particles and negative correlation with HDL particles and sub-particles (Lodge et al., 2021a), while M-CSF and IL-12p40 correlate with plasma levels of glycylproline and long-chain acylcarnitines in COVID-19 patients (Yang et al., 2022). That said, the COVID-19 interplay with the inflammatory system is not unique (Liu et al., 2022) and more than 20 metabolites were associated equally strongly to other unrelated severe pneumonia events (Julkunen et al., 2021). In line with this observation, the vast majority of tuberculosis (TB) patients experienced a severe SARS-CoV-2 post-TB infection (Diboun et al., 2022).

## Biomarkers of COVID-19 severity

From the very beginning it became clear that metabolic pathways detectable in plasma, but not other signaling pathways, could be used to stratify COVID-19 patients (Wu et al., 2020) and metabolomic analysis of several biofluids has been extensively used to discriminate patients according to severity of SARS-CoV-2 infection (Table 2). One problem is the heterogeneity in the severity criteria, but most studies abided the guidelines for diagnosis of SARS-CoV-2 issued by the WHO or by the National Health Commission of the People’s Republic of China. In Table 2, cohorts from the different studies have been unified to a single classification: asymptomatic (AS), mild (M), moderate (MO), severe (S) or critical/deceased (C) according to severity, with special classes for COVID-19 non-infected subjects (controls, CO) and people with other pathologies (other, O). Hospitalized patients are mainly S, C and O classes.

**TABLE 2:** COVID-19 severity studies.

Study	Cohort^a^	Technique^b^	Biofluid	Main metabolic markers of severity^c^
Ibarra-gonza et al. (2022)	**453**, (31CO, 152M, 60S, 210C)	MS	Serum	phenylalanine, alanine, citrulline, proline, succinylacetone
Su et al. (2020)	**397**, (258CO, 51M, 58M, 30S)	UPLC-MS	Serum	benzoate, catechol sulfate, 3-hydroxyhippurate, lipids
Sindelar et al. (2021)	**339**, (67CO, 143M/MO, 129S)	LC-MS	Serum	LPC, PC, ceramides, serine, kynerurate, 1-methyladenosine, PE
Hao et al. (2021)	**267**, (178CO, 89AS)	LC-MS	Serum	LPC, LPI, LPS, LPA, diacylglycerol, PC, PE, sphingomyelin, FAs
Pérez et al. (2022)	**254**, (46CO, 13O 36M, 50MO, 54S, 55C)	LC-MS	Plasma	acetylcholine, fatty acids/lipid mediators, arachidonic acid, linoleic acid, tetradecanoate, dodecanoate, 11-hydroxy-5Z, 8Z, 12E, 14Z-eicosatetraenoic acid), 5-hydroxy-6E, 8Z, 11Z, 14Z-eicosatetraenoic acid
Oliveira et al. (2022)	**242**, (105M/MO, 137C)	MS	Plasma	glycerophospholipid, porphyrin, linoleic acid, purine metabolism
Gu et al. (2022)	**199**, (30CO, 74M/MO, 33S, 62C)	LC-MS	Serum	20-hydroxyeicosatetraenoic acid, triethanolamine, chavicol, disialosyl galactosyl globoside, 1-arachidonoglycerophosphoinositol,α-methylstirene
Shi et al. (2021)	**187**, (78CO, 30O, 32M, 47S)	GC-MS	Serum	2-hydroxy-3-methylbutyric acid, 3-hydroxybutyric acid, cholesterol, succinic acid, ornithine, oleic acid, palmitelaidic acid, linoleic acid
Ceballos et al. (2022)	**138**, (15CO, 30O, 32M, 47S)	GC-MS and CE-MS	Plasma	tryptophan, kynurenine, 2,3-butanediol, lactic acid, citric acid, carbamate, D-glucarate, isocitric acid, lysine
Schmelter et al. (2021)	**124**, (18CO, 76O, 30S)	NMR	Serum	triglycerides, LDL, VLDL
Roberts et al. (2022)	**120**, (71M, 49S)	UPLC-MS	Serum	ureidopropionate, cytosine, kynurenine, deoxycitidine, pseudouridine, short and medium fatty acyl carnitines, pentahomomethionine, trihomomethionine, ergothioneine, piperine
Bi et al. (2022)	**115**, (27CO, 17O, 48M/MO, 23S)	LC-MS	Urine/Serum	adenosine
D’Amora et al. (2021)	**113**, (31CO, 20M, 32MO, 30S)	LC-MS	Plasma	glutamate, valeryl-carnitine, kynurenine/tryptophan citrulline/ornithine
Correia et al. (2022)	**110**, (57CO, 21M, 22MO, 10S)	NMR	Plasma	glycerol, acetate, 3-aminoisobutyrate, formate, glucuronate, lactatic acid
Danlos et al. (2021)	**101**, (29CO, 23M, 21MO, 28C)	UPLC/GC-MS	Plasma	arabinose, ribose, maltose, raffinose, arginine, aspartate, glutamate, phenylalanine, tyrosine, ornithine, spermine, spermidine, tryptophan, kynurenine metabolism, anthranilic acid, arachidonic acid, PC, PE, spingosine-1-phosphate, deoxycholic acid, trigonelline, creatine, urea
Páez-Franco et al. (2022)	**92**, (27CO, 19M, 46S)	GC-MS	Serum	α-ketoglutarate, phenylalanine, glutamate, hydroxyisovaleric acid, hydroxybutyric acid
Dillard et al. (2022)	**84**, (48M/MO, 36S)	LC-MS	Plasma	4-imidazolone-5-propanoate, 3-methylglutarylcarnitine
Rendeiro et al. (2022)	**84**, (9CO, 46M, 11MO, 18S)	NMR	Serum	albumin, HDL and small HDL particle species, cholesteryl-ester component of HDL and IDL, VLDL with increased phospholipids component and extra-small VLDL, IDL, LDL and HDL with increased triglycerides. GlycA, ApoB/ApoA1, acetoacetate, 3-hydroxybutyrate, phenylalanine
Torretta et al. (2021)	**83**, (24CO, 11M, 28MO, 12S, 8C)	UPLC-MS	Serum	sphingomyelin, acid sphingomyelinase, dihydrosphingosine, dihydroceramide, monosialodihexosyl ganglioside, dihydrosphingosine, dihydroceramide
Zhang et al. (2022)	**78**, (30CO, 39M, 9S)	LC-MS, GC-MS and CE-MS	Serum	arginine, putrescine, N-acetylputrescine, spermidine
Dewulf et al. (2022)	**75**, (19CO, 26M/MO/S, 30C)	LC-MS	Urine	tryptophan, kynurenine, 3-hydroxykynurenine, 3-hydroxyanthranilate
Xue et al. (2022)	**65**, (17CO, 40M, 8S)	UPLC-MS	Plasma	phosphatidylinositol, PC, arachidonic acid, LPC, LPE
Albóniga et al. (2022)	**63**, (36CO, 11M, 11MO, 5S)	CE-TOF-MS	Plasma	creatine, citrulline, kynurenine, tryptophan
Caterino et al. (2021a)	**61**, (9CO, 20M, 16MO, 16S)	LC/FIA-MS	Serum	lactic acid, as, glycine, aspartate, trigonelline, spermine, serotonin, succinic acid, dehydroepiandrosterone sulfate, xanthine, ornithine
Marín-Corral et al. (2021)	**49**, (13MO, 10S, 26C)	MS	Plasma	ceramides, tryptophan, kynurenine metabolism, lactate/pyruvate
Karu et al. (2022)	**44**	LC-MS	Plasma	arachidonic acid, prostanoids, lipoxygenase derivatives, linoleic acid
Wu et al. (2020)	**44**, (10CO, 14M, 11S, 9D)	UPLC-MS	Plasma	carbamoyl phosphate, guanosine monophosphate, malic acid, dihydrouracil, D-Xylulose 5-phosphate, purine metabolism

^a^ AS, asymptomatic; CO, control; M, mild; MO, moderate; S, severe; C, deceased and/or critical.

^b^ LC-MS, liquid chromatography coupled to mass spectrometry; GC-MS, gas chromatography coupled to mass spectrometry; NMR, nuclear magnetic resonance spectroscopy; UPLC, ultra-high pressure liquid chromatography; CE, capillary electrophoresis; TOF, time of flight; FIA, flow injection analysis.

^c^ LDL, low density lipoproteins; HDL, high density lipoproteins; IDL, intermediate density lipoproteins; LPC, lysophosphatidylcholine; LPE, lysophosphatidylethanolamine; LPI, lysophosphatidylinositol; LPS, ysophosphatidylserine; LPA, lysophosphatidic acid; PC, phosphatidylcholine; PE, phosphatidylethanolamine; FAs, fatty acids; VLDL, very low-density lipoproteins.

Bold value corresponds to the total number of patients in each cohort.

In an early study, Danlos et al. (2021) investigated plasma samples from a well-stratified cohort of COVID-19 patients to identify up to 77 metabolites (amino acids, polyamines, sugars and their derivatives among others) that differ between critical and mild patients. These results were further confirmed and extended by subsequent studies in plasma as well (Albóniga et al., 2022; Dillard et al., 2022), using other matrices (Buyukozkan et al., 2022; Dewulf et al., 2022) or as a part of a *multi-omic* study (Su et al., 2020; Wu et al., 2021). All these contributions highlight the critical role that kynerurate pathway has in the metabotype associated to the disease severity (D’Amora et al., 2021; Marín-Corral et al., 2021; Roberts et al., 2022). Dysregulated amino acids (Ibarra-gonza et al., 2022), alterations in intermediates of amino acid catabolism (Páez-Franco et al., 2021) and elevated porphyrins (San Juan et al., 2020; Oliveira et al., 2022) are also linked to severe phenotypes of the disease.

Not only metabolites but also lipids such as carnitines and phosphatidylcholine (Caterino et al., 2021b; D’alessandro et al., 2021; Wu et al., 2022) and an NMR-determined pro-atherogenic lipoprotein profile have been associated to COVID-19 severity (Schmelter et al., 2021; Rendeiro et al., 2022). In addition, the metabolic changes associated with severity are also correlated with immune response markers: between plasma oxylipins (Karu et al., 2022) or acetylcholine (Pérez et al., 2022) with chemokines/neutrophiles or between lysophosphatidyl choline (LPC) with IL-6 (Sindelar et al., 2021).

**FIGURE 2:**
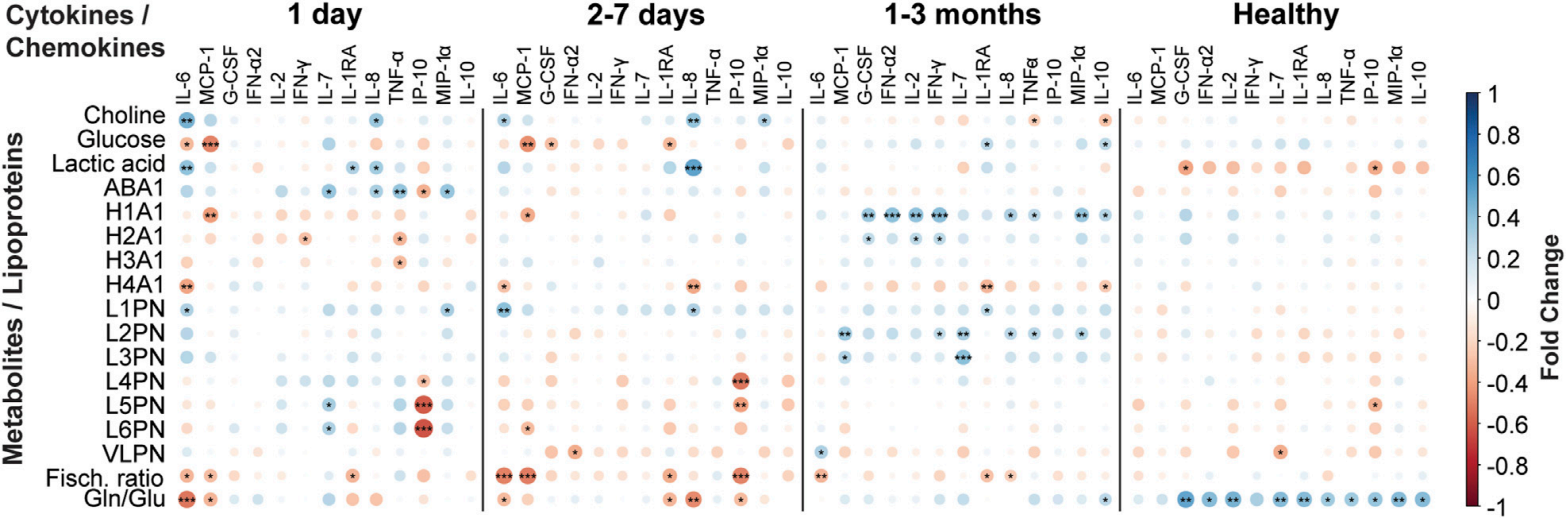
Immunometabolic correlation plots between a panel of cytokines/chemokines related with COVID-19 disease versus a set of metabolites and lipoproteins quantified in blood serum samples from COVID-19 patients. The plots represent the correlations from disease onset to up 3 months after infection. Negative correlations are coloured in red, while positive correlations are coloured in blue, as indicated in the figure legend. The calculations were performed in R.

This information has predictive power and a machine learning based model can predict COVID-19 prognosis employing only 22 plasma metabolites, most of them LPCs (Sindelar et al., 2021). Other equivalent models can be obtained when combining the information from 12 urine metabolites with proteomic data (Li T. et al., 2021), with the concerted analysis of an extended panel of lipids and metabolites with cytokines (Byeon et al., 2022) or by the combination of 21 lipids with four protein markers (Li Y. et al., 2021) (Figure 2).

## Metabonomic studies to investigate the effect of drugs

Metabonomics aims to measure the overall metabolic response of living systems to biological stimuli or to genetic manipulation (Nicholson and Lindon, 2008). In the context of COVID-19, metabonomic analyses of non-invasive samples have been widely used to investigate the effect of drugs. Spick and co-workers used serum metabonomics in combination with other *omic* techniques to investigate the mechanism of glucocorticoids in the palliation of ARDS in COVID-19 patients, which was also used as a surrogate marker for severity (Spick et al., 2022a). Consistently, the effect of corticoids or liver protective drugs (i.e., *arbitol*) could be discriminated using untargeted metabonomics, but not the combined effect of two antiviral agents (*lopinavir* and *ritonavir*), that showed similar metabotype as for untreated patients (Shi et al., 2021). Furthermore, Meoni et al. (2021) employed NMR-based metabolomics and lipidomics in plasma samples to demonstrate that treatment with *tocilizumab* partially reverts the metabolic alterations due to SARS-CoV-2 infection.

An impressive scientific and economic effort allowed the early appearance of prophylactic measures for SAR-CoV-2 infection and several studies have investigated the putative metabolic alterations induced by mRNA vaccines. Using a combination of LC-MS and NMR spectroscopy, Dagla et al. (2022) analyzed plasma samples from patients up to 3 months after the first dose and observed distinct plasma metabotypes in relation to the level of immune response, highlighting the role of amino acid metabolism and the lipid profile as predictive markers of response to vaccination (Dagla et al., 2022). Equivalent variations in the lipoproteins (but not in the metabolome) were observed by serum NMR profiling of vaccinated individuals (Ghini et al., 2022a). In turn, He et al. (2022) have investigated the serum metabolic profiles associated with a proper response of the host immune antibodies and cytokines. Finally, the effect of nutrition as a protective factor for COVID-19 prognosis has also been investigated by lipidomic analysis (Barberis et al., 2021b).

## Metabolic recovery of COVID-19 patients

Metabolomics has been extensively used to investigate the natural history of COVID-19 and patient’s recovery at the metabolic level. Several studies have investigated the metabolic recovery of COVID-19 patients by analyzing blood (serum or plasma) of patients from onset to discharge from hospital and the subsequent follow up check-ups, spanning up to more than 1 year from the disease onset (Table 3). Characterization of metabolic phenoreversion, a concept introduced by Jeremy Nicholson in the context of COVID-19 to describe its metabolic evolution (Lodge et al., 2021b), reveals that partial reversion of the metabolic phenotype can be associated to severity of the disease (Holmes et al., 2021a), and it correlates with an abnormal pulmonary function after three (Xu et al., 2021) or even six (Li H. et al., 2022) months from the disease onset. Other studies also suggest a slower metabolic phenoreversion as compared to the patient’s discharge time (Zhang et al., 2021), with dysregulated lipoprotein profile after hospital discharge (Bizkarguenaga et al., 2022). Persistent alterations of the metabolism concentrate in the amino acids, organic acids, purine, fatty acids (Valdés et al., 2022) and lipid metabolism (Kurano et al., 2022a), while the kynurenate pathway returned to normal levels (Li F. et al., 2022).

**TABLE 3:** COVID-19 metabolic recovery and natural history studies.

Study	Cohort^a^	Technique^b^	Biofluid	Time period	Main metabolic markers^c^
Valdés et al. (2022)	**145**, (25CO, 28RAS, 27RM, 36RS, 29RC)	LC-MS	Plasma	2–3 months	LPC, phenylacetyl-l-glutamine, bilirubin, L-methionine, hypoxanthine, inosine, acetaminophen sulfate, myclobutanil, nervonic acid, 1-methyladenosine, L-tryptophanamide, methylsuccinic acid, octadecanedioic acid
Bizkarguenaga et al. (2022)	**140**, (71CO, 69RMO/RS)	NMR	Plasma	3–10 months	porphyrins, free and total cholesterol, LDL, HDL
Zhang et al. (2021)	**135**, (39CO, 18RAS, 34RMO, 44RS/RC)	LC-MS	Plasma	3 months	taurine, succinic acid, hippuric acid, amino acids, bile acids, organic acids, indolelactate, cyclic AMP, citric acid, lactoylglutathione, ribitol
Xu et al. (2021)	**130**, (27CO, 34RM/RMO, 69RS/RC)	LC-MS	Plasma	3 months	acetyltyrosin, betaine, glycerophospholipid, triacylglycerols, taurine, PC, prostaglandin E2, arginine, adenosine, acylcarnitine, fatty acids, hypotaurine α-linolenic acid, epoxyeicosatrienoic acid, palmitoleic acid
Li et al. (2022b)	**84**, (27CO, 34RM/RMO, 69RS/RC)	UPLC/LC-MS	Plasma	6 months	apolipoproteins, lipids
Liptak et al. (2022)	**62**, (37CO, 25RS)	NMR	Plasma	1 month	histidine, creatinine, succinate, glucose, lipoproteins, 3-hydroxybutyrate
Li et al. (2022a)	**62**, (22CO, 22RM/RMO)	LC-MS	Serum	6 months	amino acids, organic acids, purine, fatty acids, and lipid metabolism

^a^ RAS, recovered asymptomatic; CO, control; RM, recovered mild; RMO, recovered moderate; RS, recovered severe; RC, recovered critical.

^b^ LC-MS, liquid chromatography coupled to mass spectrometry; GC-MS, gas chromatography coupled to mass spectrometry; UPLC, ultra-high pressure liquid chromatography; NMR, nuclear magnetic resonance spectroscopy.

^c^ AMP, adenosine monophosphate; LDL, low density lipoproteins; HDL, high density lipoproteins; LPC, lysophosphatidylcholine; PC, phosphatidylcholine.

Bold value corresponds to the total number of patients in each cohort.

**FIGURE 3:**
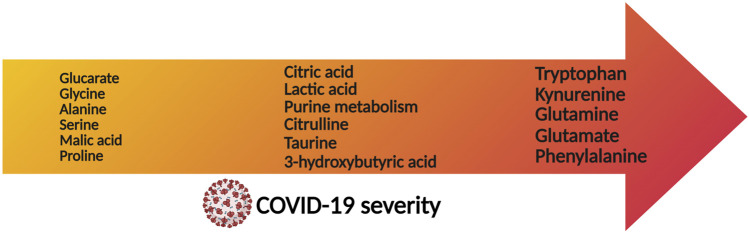
Metabolites that are associated with severity prognosis of COVID-19, sorted according to the disease severity (arrow axis).

Prospective observational studies have proven useful to investigate the natural history of metabolic phenoreversion (Liptak et al., 2022). These studies evidence the intimate relationship between metabolic phenoreversion and the normalization of the exacerbated immune response (Jing et al., 2022; Zhang et al., 2022). In a large prospective study (Table 3), acute patients showed a metabolic and lipidomic dysregulation that accompanies the exacerbated immunological response, resulting in a slow metabolic recovery time with a maximum probability around 62 days (unpublished data). As an example, Figure 3 shows the correlation between a panel of COVID-19 associated inflammatory markers as compared to disease-altered metabolites and lipoproteins, as a function of the recovery time. The slow metabolic normalization in acute patients is lineage dependent (Lewis et al., 2022) and it maintains for months a lipoprotein profile compatible with enhanced atherosclerotic risk (unpublished data), providing an explanation for the elevated number of cardiovascular episodes found in postCOVID-19 cohorts (Xie et al., 2022). In line with this idea, survivors from non-severe COVID-19 from Wuhan still show metabolic abnormalities after 6 months (Li F. et al., 2022). This is consistent with previous studies on other related viruses such as MERS and SARS-CoV-1.

Unfortunately, COVID-19 does not always evolve towards a full restoration of the metabolism and post-acute sequelae of COVID-19 represent an emerging global crisis (Su et al., 2022) and the need to find biomarkers for long COVID is pressing. In this context, deconvolution of NMR spectra from COVID-19 sera identified three diagnostic subregions of the supramolecular phospholipid composite signal envelope that provide insight about the increased cardiovascular risk in COVID-19 patients and the risk persistence in post-acute COVID-19 syndrome (Masuda et al., 2022). Notably, lipoproteins emerge as an important prognostic biomarker for the prediction of long COVID effects (Bai et al., 2021).

## Discussion and concluding remarks

SARS-CoV-2 infection produces a profound metabolic dysregulation that can be adequately characterized by metabolomics and lipidomics. That said, the different techniques used, and the inherent variability impede a proper comparison between studies, particularly when considering the quantification of the changes associated to COVID-19. Many altered metabolites and lipoproteins can be used to evaluate disease severity and to monitor drug intervention, with a subset of biomarkers that also show prognostic value to evaluate long-term sequelae of the disease. The long-standing dysregulation of lipoprotein metabolism in COVID-19 patients provide an explanation for the elevated risk of cardiovascular episodes detected in post-infection individuals.

## Footnotes

1
https://covid19.who.int.
